# Facile Fabrication of Nickel Supported on Reduced Graphene Oxide Composite for Oxygen Reduction Reaction

**DOI:** 10.3390/nano13243087

**Published:** 2023-12-05

**Authors:** Yanan Wang, Jianhua Qian, Junhua Li, Jinjuan Xing, Lin Liu

**Affiliations:** 1 School of Materials Science and Engineering, Northeastern University, Shenyang 110819, China; wynants@163.com; 2 School of Petrochemical Engineering, Liaoning Petrochemical University, Fushun 113001, China; 3 Liaoning Key Laboratory for Surface Functionalization of Titanium Dioxide Powder, College of Chemistry and Materials Engineering, Bohai University, Jinzhou 121013, China

**Keywords:** reduced graphene oxide, metal–support interaction, oxygen reduction reaction, solid-phase spillover, microwave-assisted hydrothermal method

## Abstract

Due to the depletion of fossil fuels, the demand for renewable energy has increased, thus stimulating the development of novel materials for energy conversion devices such as fuel cells. In this work, nickel nanoparticles loaded on reduced graphene oxide (Ni/rGO) with small size and good dispersibility were successfully prepared by controlling the pyrolysis temperature of the precursor at 450 °C, assisted by a microwave-assisted hydrothermal method, and exhibited enhanced electrocatalytic activity towards oxygen reduction reaction (ORR). Additionally, the electron enrichment on Ni NPs was due to charge transfer from the rGO support to metal nickel, as evidenced by both experimental and theoretical studies. Metal–support interactions between nickel and the rGO support also facilitated charge transfer, contributing to the enhanced ORR performance of the composite material. DFT calculations revealed that the first step (from O_2_ to HOO*) was the rate-determining step with an RDS energy barrier lower than that of the Pt(111), indicating favorable ORR kinetics. The HOO* intermediates can be transferred onto rGO by the solid-phase spillover effect, which reduces the chemical adsorption on the nickel surface, thereby allowing continuous regeneration of active nickel sites. The HO_2_^−^ intermediates generated on the surface of rGO by 2e^−^ reduction can also efficiently diffuse towards the nearby Ni surface or the interface of Ni/rGO, where they can be further rapidly reduced to OH^−^. This mechanism acts as the pseudo-four-electron path on the RRDE. Furthermore, Ni/rGO-450 demonstrated superior stability, methanol tolerance, and durability compared to a 20 wt% Pt/C catalyst, making it a cost-effective alternative to conventional noble metal ORR catalysts for fuel cells or metal–air batteries.

## 1. Introduction

As fossil fuels become depleted, the development of new energy sources is imperative. Fuel cells are the most promising electrochemical energy conversion devices and directly convert the chemical energy of fuels and oxidants into electrical energy with high efficiency and low environmental impact [1]. However, the development of commercial-fuel-cell noble metal catalysts has been severely limited by cost, durability, and methanol tolerance issues [2,3]. Therefore, to minimize the cost and consumption of noble metals to the greatest extent, non-noble transition metal-based catalytic materials have been explored [4,5,6,7,8]. In our study, metallic nickel was chosen as an alternative to the noble metal platinum for investigation. Nickel has good electron transfer capabilities, which facilitates improved electron transfer efficiency for the oxygen reduction reaction. The d-orbital energy level of metallic nickel matches well with the π* orbital of oxygen molecules, which is beneficial for the adsorption and activation of oxygen molecules, thereby enhancing the ORR catalytic performance. Nickel-based catalysts also demonstrate good tolerance to common poisons like sulfides and alkali metal ions. Thus, they possess good corrosion resistance in alkaline media, enabling their preserved stability and prolonged service life. Additionally, nickel reserves are abundant, ranking fifth in the Earth’s crust after silicon, oxygen, iron, and magnesium, and relatively low in cost, making nickel more competitive for large-scale applications [9].

Carbon-based materials are common metal electrocatalyst supports used in fuel cells, playing an important role in catalyst performance [10,11,12]. Not only do they support metal nanoparticles (NPs), but they also facilitate electron/mass transfer and stability through metal–support interactions (MSI) [13]. Among them, graphene materials possess high specific surface areas, excellent conductivity, and adequate porosity [14,15]. They can achieve uniform dispersion of metal NPs through high electrochemically active surface areas, thereby maximizing the utilization of active metals and providing more active sites for catalytic reactions. Hence, they are quite promising among various catalyst supports [16].

More importantly, interface sites surrounding metal NPs represent a unique environment since they are in direct contact with metal NPs, supports, and reactants, thereby promoting catalytic reactions synergistically. In addition, interface-site atoms have been demonstrated to facilitate accumulation of excess charges during charge transfer [17]. All these can significantly enhance the adsorption and reaction of oxygen molecules and intermediates on interface sites. Moreover, the intimate vicinity of metal NPs to different groups or defects (e.g., oxygen vacancies, hydroxyls, etc.) on the support surface may also contribute to the local sequential reaction of reactants or products or may stabilize the transition state [10]. Therefore, we chose reduced graphene oxide (rGO) with partial surface defects as the support. Spillover across the interface periphery may also occur, initiated at one surface, usually the metal NPs, activating a reactant and then transferring it to a support surface that does not activate the reactant itself under the same conditions. The most studied spillover is that of hydrogen as well as oxygen intermediates or other molecules. Solid-phase spillover effects help reduce the chemical adsorption of reactive intermediates on a metal surface, enabling continuous regeneration of active sites on metal surfaces [18].

Hence, it is crucial to optimize those calcination conditions controlling the size of the metal particles while ensuring their high dispersion and effective charge transfer. This approach enhances the synergy between the metal and carbon carrier and improves the overall catalytic efficiency. In this work, a simpler green synthesis method involving a microwave-assisted hydrothermal process assisted by pyrolysis temperature control of the precursor was adopted to successfully prepare reduced graphene oxide (rGO)-supported nickel NPs (Ni/rGO) with small size and good dispersion. The as-prepared optimum Ni/rGO (Ni/rGO-450) has good catalytic activity for electrocatalytic oxygen reduction reactions and demonstrates excellent long-term stability, durability, and anti-poisoning properties compared to commercial 20 wt% Pt/C. Therefore, it can replace expensive commercial Pt/C catalysts at a low cost.

## 2. Fabrication Process of Ni/rGO Composite

**Figure 1:**
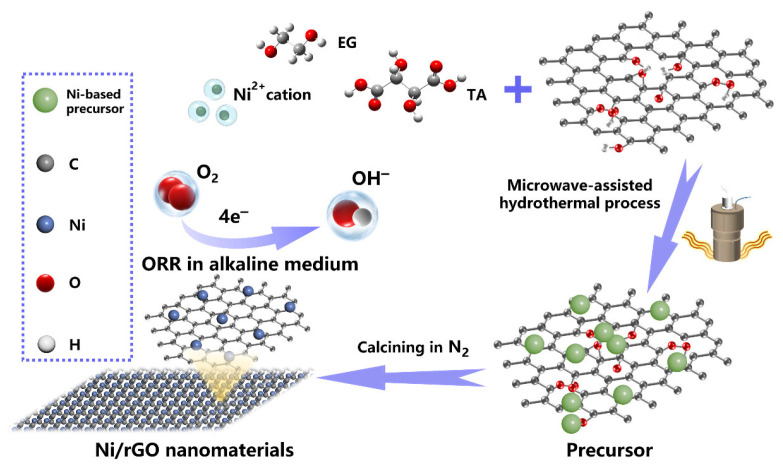
Schematic illustration of Ni/rGO prepared by a microwave-assisted hydrothermal treatment method.

A schematic diagram of the entire formation process of Ni/rGO as prepared by the microwave-assisted hydrothermal treatment method is shown in Figure 1. After annealing in the N_2_ atmosphere, the Ni-based precursor on the surface of rGO was further reduced to form Ni particles. The samples under different calcination temperatures in the range of 350–800 °C are denoted as Ni/rGO-T. All the materials used in the fabrication of Ni/rGO and the fabrication process details are provided in the Supplementary File. The physical and electrochemical measurement details and the calculation details are also given in the Supplementary Materials.

## 3. Results and Discussion

### 3.1. Morphology, Phase, and Element Composition Analysis

**Figure 2:**
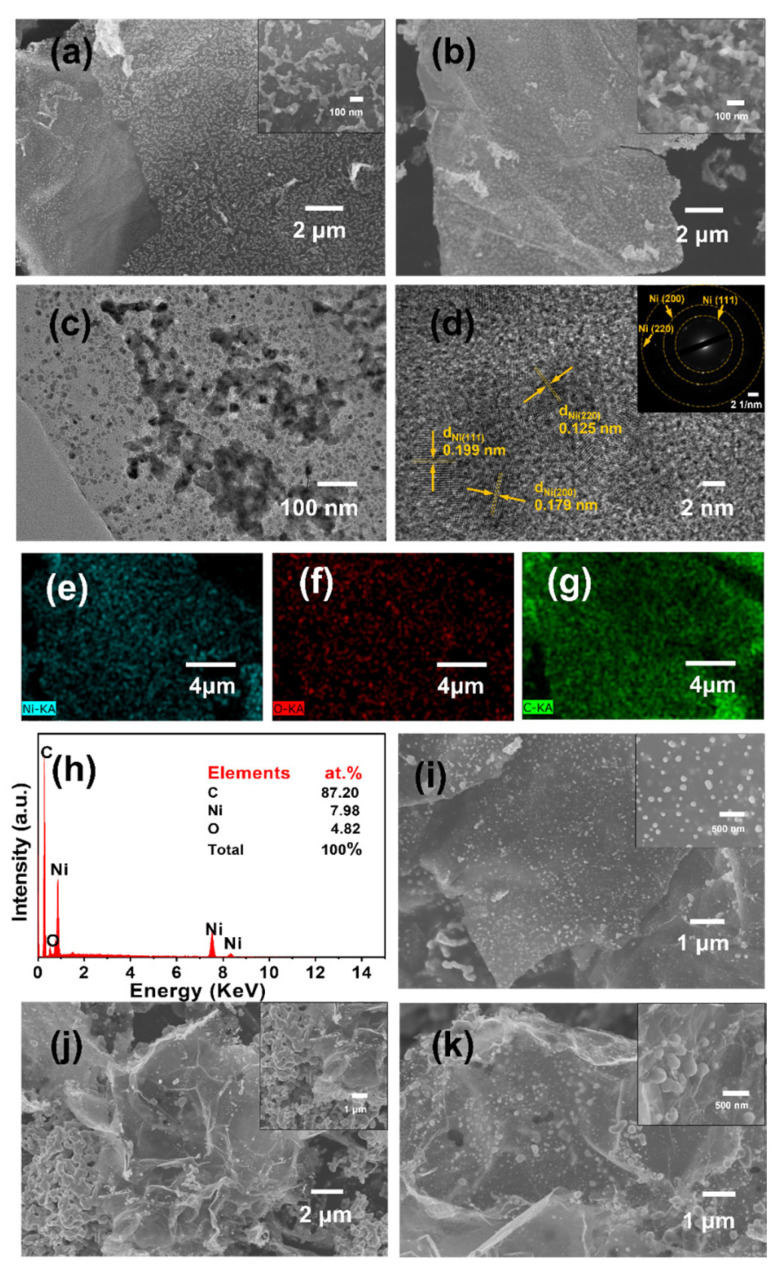
SEM images of Ni/rGO at different calcination temperatures: (**a**) Ni/rGO-350, (**b**) Ni/rGO-450, (**i**) Ni/rGO-550, (**j**) Ni/rGO-650, and (**k**) Ni/rGO-800; (**c**) TEM image, (**d**) HRTEM image, (**e**–**g**) elemental mapping images of Ni, O, and C and (**h**) EDS of Ni/rGO-450.

SEM images of the prepared Ni/rGO at different calcination temperatures are shown in Figure 2a,b,i–k. As can be seen, the Ni particles on the rGO support gradually became larger and more agglomerated as the calcination temperature rose. The Ni NPs in Ni/rGO-450 had a much narrower particle size distribution as shown in Figure 2b, with a mean particle size of approximately 15 nm. Elemental mapping images in Figure 2e–g also showed that the Ni NPs in the Ni/rGO-450 composite were well dispersed on the rGO. In addition, the N_2_ adsorption and desorption curves of the precursor and Ni/rGO composites at different calcination temperatures are shown in Figure S2. The specific surface area of the Ni/rGO composites after calcination decreased compared to that of the precursor, as shown in Table S1. However, the specific surface area of Ni/rGO-450 was greater compared to that of other Ni/rGO composites at different calcination temperatures. This is due to the small particle size and uniform distribution of Ni NPs without agglomeration, which facilitates maximum exposure of the surface, thereby favoring the exposure of more catalytically active sites. Moreover, it can be observed that three lattice spacings of 0.125 nm for (220), 0.199 nm for (111), and 0.179 nm for (200) can be assigned to the crystal planes of Ni particles from the high-resolution TEM image shown in Figure 2d [19]. The selected SAED pattern, shown as an inset in Figure 2d, depicts ring fringes generated by the polycrystalline properties of Ni. Figure 2h displays the Ni loading of Ni/rGO-450, which was 7.98%. The high metal dispersion in Ni/rGO-450 implies a potent interaction between the metal and the support, which hinders clustering into large aggregates [20]. The strong interaction is probably due to the C=O double-bond functional groups on graphene sharing electron pairs with the empty orbitals of the metal Ni [21]. The above results highlight the advantageous impact of using rGO as a catalyst support, as evidenced by the effective metal dispersion observed.

**Figure 3:**
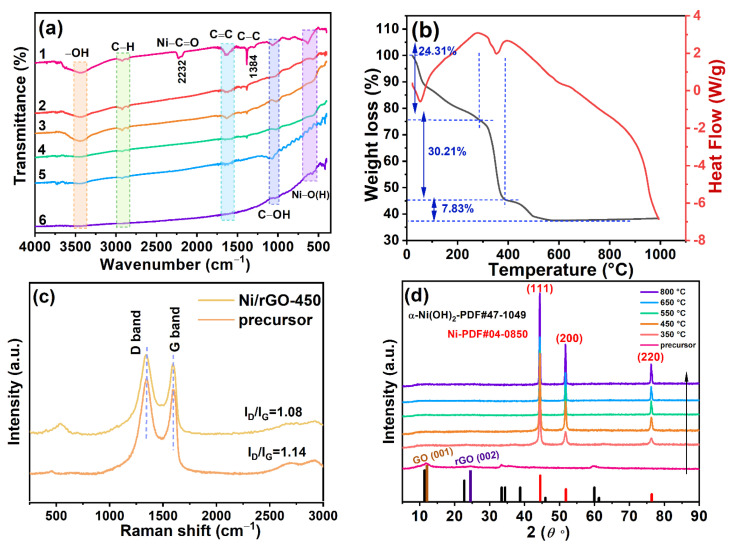
(**a**) FTIR spectra of precursor and Ni/rGO at different calcination temperatures of (1) precursor, (2) Ni/rGO-350, (3) Ni/rGO-450, (4) Ni/rGO-550, (5) Ni/rGO-650, and (6) Ni/rGO-800; (**b**) TG–DTA curves of the precursor; (**c**) Raman spectra of precursor and Ni/rGO-450; (**d**) XRD patterns of precursor and Ni/rGO at different calcination temperatures.

As shown in the FTIR spectra (Figure 3a), a broad peak resulting from adsorbed water was observed in all samples at about 3443 cm^−1^ [22]. For sample 1, the peak detected at ~2232 cm^−1^ was attributed to the carbonyl band of a metal carboxylate stretch (carboxyl group of tartaric acid coordinated to Ni) [23]. The other observed peaks, namely those at ~1058 cm^−1^ and ~2921 cm^−1^, were identified as the tensile vibrations of C–OH and C–H of the rGO, respectively [24,25]. The peaks of all samples at 1636 cm^−1^ and 1384 cm^−1^ were assigned to the C=C and C–C skeletal vibrations of unoxidized graphite [26], respectively. The peak intensity of the C–C skeleton at 1384 cm^−1^ weakened, indicating an improvement in the degree of graphitization of the precursor after sintering, accompanied by the conversion of the GO to rGO. The peak of the precursor at 626 cm^−1^ was caused by the stretching and bending vibration of Ni–OH [27]. For samples 2 to 6, the peak corresponding to Ni–O(H) appeared with a redshift, indicating that the metal hydroxide gradually dissociated and dehydrogenated with an increase in calcination temperature so that Ni nanoparticles combined with oxygen on the surface of the graphene substrate to form Ni–O bonds. As depicted in Figure 3b, 24.31% of the total mass of the Ni-based precursor was lost between 25 °C and 288 °C. This was due to the removal of residual H_2_O, unpolymerized TA, and other reactant molecules, with an endothermic peak at 55 °C. Between 288 °C and 385 °C, 30.21% of the entire mass reduction was attributed to the degradation of nickel-based hydroxide, Ni–glycolic acid, Ni_2_(OH)_2_CO_3_, and so on, corresponding to an exothermic peak at about 288 °C. Meanwhile, the GO was further reduced to rGO, which corresponded to the endothermic peak at 355 °C [28]. The loss of 7.83% of the total weight was attributed to the carbonization of the carbon-containing precursor and the reduction of amorphous Ni compounds to Ni NPs combined with rGO in the temperature range of 385 °C to 560 °C. FTIR and TG–DTA analysis confirmed that with an increase in calcination temperature, metal hydroxides, metal complexes, and other components in the precursor gradually decomposed, and Ni NPs formed Ni–O bonds with rGO, achieving a stable interfacial combination [29].

As observed in the Raman spectra (Figure 3c), two significant peaks at 1346 cm^−1^ and 1591 cm^−1^ are present, corresponding to the D peak (representing lattice defects of the C atom) and the G peak (representing the in-plane tensile vibration of the sp2 C atom) [30]. The reduction in peak D’s strength, as observed in Figure 3c, directly indicates a decrease in the sp3 hybrid structure. The reduction–oxidation degree of GO can be assessed by the I_D_/I_G_ ratio, which represents the intensity ratio between the D peak and the G peak [31,32]. The smaller the I_D_/I_G_ value, the higher the reduction degree of GO and the lower the oxidation degree of graphite and vice versa [33]. From Figure 3c, it is evident that the Ni/rGO sample exhibits a lower I_D_/I_G_ value (1.08) compared to its precursor (1.14), indicating an increased reduction degree of GO. The peak observed at approximately 531 cm^−1^, attributed to the vibrational peaks of Ni–O [34], signifies the successful anchoring of Ni NPs on the graphene sheets in the as-synthesized Ni/rGO catalysts. Raman results showed that with an increase in temperature, GO underwent further reduction, the degree of oxidation decreased, and defects reduced, which is beneficial to enhancing the interaction between Ni NPs and rGO substrate. Comprehensively, the above characterization analysis revealed that with an increase in temperature, Ni–O bonds formed at the metal–support interface where the electron cloud was reconstructed, promoting charge transfer. This interfacial regulation mechanism may enhance the catalytic activity of Ni. The stable anchoring of Ni NPs on rGO helps inhibit the oxidation and aggregation of metallic nickel. By optimizing the calcination temperature to achieve highly dispersed Ni and controlled surface functional groups of rGO, it is key to obtain a synergistic effect, which provides an effective strategy for designing high-performance non-noble metal catalysts.

As displayed in Figure 3d, the precursor displayed distinctive peaks at 12.0°, 33.2°, and 59.3° corresponding to the (003), (101), and (110) planes of α-Ni(OH)_2_ (JCPDS 47-1049) [35], respectively. The peaks at 11.8° and 24.3° correspond to the (001) and (002) planes of GO and rGO, respectively. With an increase in annealing temperature, the peak intensity of α-Ni(OH)_2_ and GO decreased gradually and no α-Ni(OH)_2_ peak was found in the Ni/rGO annealed at temperatures greater than 350 °C. This trend indicated that α-Ni(OH)_2_ was gradually transformed into a face-centered cubic metal Ni phase after annealing. Figure 3d also highlights the significant characteristic peaks of the Ni phase (JCPDS 04-0850) [36] in all Ni/rGO composites at 44.4°, 51.7°, and 76.3°, which corresponded to (111), (200), and (220) planes, respectively [29], indicating that nickel ions were reduced to metallic Ni NPs, and no impurities were precipitated. Thus, Ni/rGO nanocomposites were successfully formed. The calcination stage is crucial for the crystallization and growth of grains. The XRD patterns of the as-prepared Ni/rGO at different calcination temperatures were further analyzed. According to the Debye–Scherrer equation (corresponding to Equation (1)), the average grain size of Ni particles on the rGO was calculated. The results are shown in Table 1.

(1) $$D\;=\;\frac{k\lambda }{\beta \cos\theta }$$

where *D* represents the grain size, *k* is the shape factor (0.9 for spherical particles), *λ* represents the wavelength of X-rays (Cu Kα = 0.15418 nm), *β* represents the full width at half maximum (FWHM) of an individual peak at 2*θ*, and *θ*(2*θ*/2) denotes the Bragg reflection angle. The calculated particle sizes are shown in Table 1. From the calculation results presented in Table 1, it is evident that the mean crystallite size of Ni NPs gradually increased with an increase in calcination temperature. This is consistent with the results of SEM. According to the theory of grain growth kinetics, the grain size is exponentially related to the reciprocal of the annealing temperature (1/*T*) (corresponding to Equation (2)) [37].

(2) lnD=-Q/RT+lnA

where *D* represents the grain size, *Q* is the activation energy for crystal nucleus growth, and *T* is the annealing temperature. ln*D* was plotted against 1/*T* and a linear fit was performed as shown in Figure S3. The temperature at 550 °C was taken as the boundary point, and the activation energy for crystal nucleus growth calculated by segments was taken as *Q*_1_ = 7.51 kJ mol^−1^ at calcination temperatures below 550 °C and *Q*_2_ = 10.79 kJ mol^−1^ at calcination temperatures above 550 °C. It was observed that Ni particle growth was faster at calcination temperatures exceeding 550 °C and slower at higher temperatures. The activation energies for crystal nucleus growth for the two temperature ranges were relatively small, so the formation of Ni nanoparticles was mainly by surface diffusion [38]. In summary, appropriate calcination temperatures can promote the diffusion and migration of metal atoms towards vacancies in the support material, such as the vacancies in graphene, allowing the metal to embed within the support structure and form MSI. However, high calcination temperatures can lead to agglomeration and growth of metal NPs, decreasing the metal–support contact area and weakening the MSI, which is detrimental to catalytic performance.

**Figure 4:**
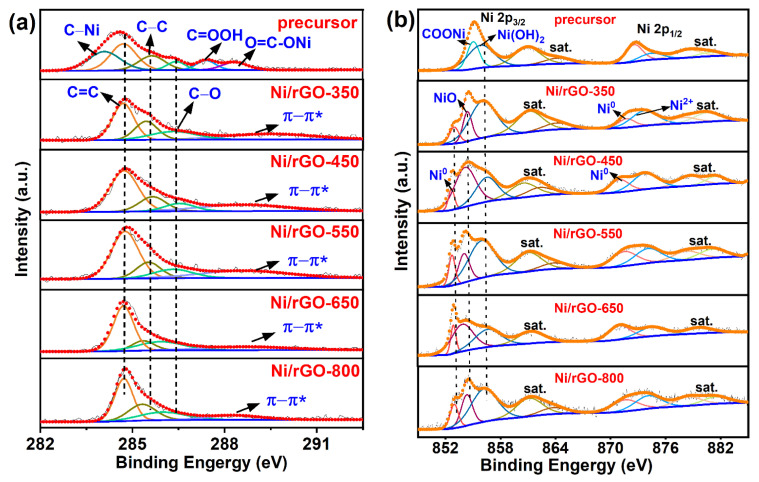
XPS high-resolution element spectra of (**a**) C 1s and (**b**) Ni 2p of precursor and Ni/rGO catalysts at different calcination temperatures.

The XPS survey of the precursor and Ni/rGO at different calcination temperatures is shown in Figure S4a, which indicates that Ni, O, and C are found in all of the samples and that there is N in the precursor but not in all Ni/rGO samples, indicating that N does not participate in the coordination after calcining. The full spectrum was corrected by C 1s binding energy (284.6 eV). The fitted O 1s spectra of each sample are presented in Figure S4b. The peak at 530.0 eV in the precursor corresponded to Ni–OOC, consistent with the fitting results of Ni 2p and C 1s, indicating that there was indeed a Ni-based coordination precursor in the precursor. The peak at a binding energy = 529.25 eV of the Ni/rGO was assigned to the lattice oxygen of the NiO lattice [39]. However, there is no diffraction peak of the corresponding oxidized species in the XRD patterns (Figure 3d), because there are relatively few oxidized species, which cannot be observed by XRD. The peak at ~530.90 eV of all samples was considered as the signal of the Ni–O–C bond [40], which indicated that the surface oxygen of the rGO matrix was closely associated with nickel species. The Ni/rGO samples all contained –OH(~532.75 eV) [41], which was attributed to adsorbed water, and Ni–OH bonds [42] (532.25~531.75 eV) in the fitted peaks. However, the Ni–OH bond disappeared in Ni/rGO-800. This may be because the calcination temperature is so high that the adsorbed molecules on the Ni surface are removed. The C 1s spectra in Figure 4a showed that the peaks of the precursor at 284.14, 284.78, 285.72, 286.43, 287.40, and 288.38 eV were attributed to C–Ni, C=C, C–C C–O, –C=OOH and –COONi coordination bonds, respectively [22,43,44]. With an increase in calcination temperature, the peak intensity related to oxygen functional groups in Ni/rGO decreased significantly and finally gradually disappeared, indicating that the oxygen functional groups of GO were effectively removed after heat treatment. Meanwhile, the C=C peak shifted to the high-binding-energy direction and showed a π–π* peak belonging to graphene at 288.25 eV [45]. Two distinct splitting peaks of Ni 2p_1/2_ and Ni 2p_3/2_ are displayed in Figure 4b owing to spin–orbit coupling. Similarly, the corresponding satellite peaks were acquired as a result of the multiple splitting of spin–orbit energy levels. In Figure 4b, it is evident that the precursor’s Ni comprised a Ni complex (–COONi, 854.4 eV) [46] and Ni(OH)_2_ (856.0 eV). As the precursor was calcined, the characteristic peak of Ni^0^ (~851.95 eV) [46] and NiO (854.40 eV) began to appear in Ni/rGO-350 and the characteristic peak of –COONi gradually disappeared. In the high-resolution XPS Ni 2p spectra of the precursors and Ni/rGO catalysts, there was a negative shift in the binding energy of Ni^0^ and a characteristic peak of NiO that indicated the enrichment of electrons on Ni NPs. This enrichment is attributed to the charge transfer effect from rGO to metallic nickel [10], which can enhance the electron density of the active sites on the metal catalyst, thus strengthening the interaction between the metal surface and the reactants [47], promoting the oxygen reduction reaction.

### 3.2. ORR Performance Analysis

**Figure 5:**
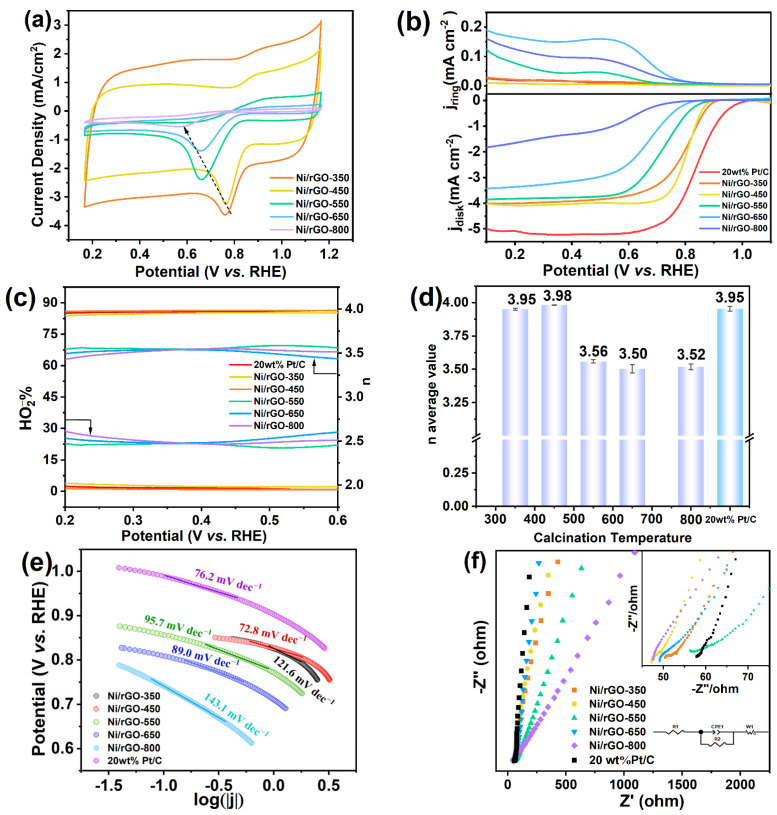
(**a**) CV curves of the Ni/rGO catalysts in O_2_-saturated 0.1 mol L^−1^ KOH at a scan rate of 50 mV s^−1^, (**b**) LSV curves, (**c**) H_2_O_2_ selectivity, and (**d**) electron transfer numbers of different as−fabricated Ni/rGO catalysts; (**e**) Tafel plots and (**f**) Nyquist plots of different fabricated Ni/rGO catalysts.

The electrocatalytic ORR activity of Ni/rGO was further tested. As illustrated in Figure 5a, all of the fabricated samples displayed distinct cathodic peaks for ORR in O_2_-saturated CV curves, while no discernible peaks were observed in N_2_-saturated CV curves, as demonstrated in Figure S5(a1–a5). The oxygen reduction peak of the Ni/rGO-450 catalyst appeared at 0.766 V (vs. RHE), which was more positive than that of other Ni/rGO catalysts, indicating that the overpotential was smaller and the oxygen reduction reaction occurred more easily. To further explore the catalytic activity of ORR, the LSV curves of the disk and the ring disk for the Ni/rGO composites modified on the RDE or RRDE electrode are compared in Figure S5(b1–b5) and Figure 5b. When the calcination temperature was 450 °C, the maximum diffusion current density at 1600 rpm of Ni/rGO-450 was 4.04 mA cm^−2^. Its onset potential and half-wave potential (E_1/2_) were 0.864 V and 0.800 V, respectively, which were close to those of the commercial 20 wt% Pt/C catalyst (E_onset_ = 0.987 V, E_1/2_ = 0.822 V, as shown in Figure S6b). In addition, a small amount of peroxide was produced during the ORR reaction. According to the RRDE test results and Equations (S2) and (S3), the yield of H_2_O_2_ and the electron transfer number were calculated as displayed in Figure 5c,d. Figure 5c shows that the hydrogen peroxide yield for Ni/rGO-450 in the voltage interval of 0.2~0.6 V was less than 5%, which is much lower than that of other Ni/rGO catalysts. As can be calculated from Figure 5d, the average electron transfer number for the ORR of Ni/rGO-450 was 3.98 which was closer to 4 than that of 20 wt% Pt/C (*n* = 3.95) shown in Figure S6b. This suggested that Ni/rGO-450 was the most active catalyst for ORR. According to the Sabatier principle, the combination of key reaction intermediates and catalysts should be neither too strong nor too weak for the catalysts to play their best role. We expect to achieve the best electronic interaction between oxygen-containing intermediates and active sites with Ni/rGO-450, thus promoting ORR kinetics. To further study the mechanism and kinetic properties of catalytic materials for ORR, the Tafel slope (Figure 5e) was obtained from the LSV data. At a higher overpotential, the Tafel slope of Ni/rGO-450 (72.8 mV dec^−1^) was close to that of commercial 20 wt% Pt/C (76.2 mV dec^−1^), indicating a more efficient ORR pathway and a similar rate-determining step during ORR [48]. In addition, a smaller Tafel slope means a faster kinetic process, indicating that the Ni/rGO-450 catalyst can reach the required current at a lower overpotential [49]. Based on the above analyses, Ni/rGO-450 exhibited favorable oxygen reduction performance. This is attributed to the oxygen reduction reaction (ORR) activity of the catalyst being influenced by the metal–support interaction (MSI) strength. In the Ni/rGO-450 composite catalytic material, the metallic nickel NPs have smaller grain sizes and are uniformly and effectively dispersed on the single-layer rGO support with less particle aggregation. Therefore, the stronger MSI improves the electron transfer efficiency, further enhancing the reaction kinetics, thus conferring enhanced catalytic activity [16].

Electrochemical impedance spectroscopy (EIS) was employed to examine the dynamic behavior of both bound and mobile charges within the bulk or interfacial regions of any given solid or liquid material [50], as shown in Figure 5f. The inset in Figure 5f shows the magnification of the high-frequency Nyquist plots and the fitted equivalent circuit. The equivalent circuit model consists of R_1_ in series, R_2_ in parallel with a constant phase element (CPE), and W_o1_ in series. R_1_ is the resistance of the electrolyte solution (R_s_). R_2_ is the charge transfer resistance (R_ct_) at the Ni/rGO–electrolyte contact interface. CPE_1_ represents a constant phase element used as a substitute for ideal capacitors/resistors that account for non-ideal conditions at the interface, which reflects uneven and defective surfaces at the catalyst/solution interface [51]. The Warburg impedance (W_o1_) was attributed to the semi-infinite diffusion of OH^−^ ions on the electrode surface. The semicircular diameter in Nyquist plots in higher-frequency regions represents charge transfer resistance (Rct). For the optimum Ni/rGO-450 electrode, the R_2_ value was smaller than that of other Ni/rGO samples and 20 wt% Pt/C. This suggests that the optimum Ni/rGO-450 composite had superior electron transport ability, which is a crucial advantage for ensuring excellent ORR activity [52]. This is because the more effective hybrid coupling between Ni metal particles and π electrons of graphene can form a smaller contact resistance. The slope of the Ni/rGO-450 electrode is above 45°, suggesting that the electrode exhibits a superior electrolyte ion diffusion behavior.

**Figure 6:**
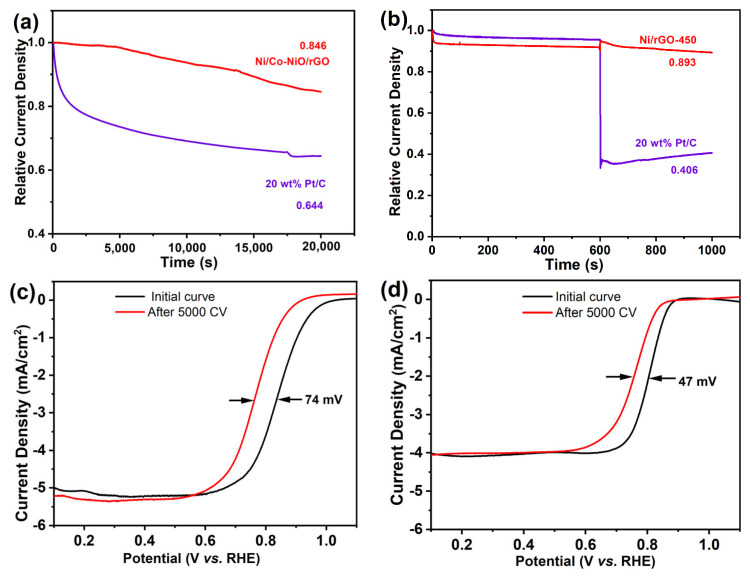
(**a**) Chronoamperometric (I–t) curves and (**b**) the methanol tolerance test curves of the Ni/rGO-450 and 20 wt% Pt/C in O_2_-saturated 0.1 mol L^−1^ KOH at 1600 rpm; LSV curves of (**c**) 20 wt% Pt/C and (**d**) Ni/rGO-450 before and after 5000 cycles of CV in O_2_-saturated 0.1 mol L^−1^ KOH at 1600 rpm.

The stability and methanol poisoning resistance of Ni/rGO catalyst and commercial 20 wt% Pt/C were also investigated by chronoamperometry (I–t). Figure 6a shows that the relative current density of the Ni/rGO-450 catalyst after a 20,000 s reaction was 0.846 compared to 0.644 of 20 wt% Pt/C, indicating that Ni/rGO has better long-term stability than commercial 20 wt% Pt/C. Additionally, the resistance to methanol poisoning must be considered when selecting an ORR catalyst for fuel cells. This is due to methanol molecules (CH_3_OH) being able to effortlessly pass through anodes to cathode membranes [53], thus diminishing the ORR activity of cathode catalysts. The effects of methanol on Ni/rGO and 20 wt% Pt/C catalysts were investigated by plotting the I–t curves in 0.1 M KOH containing 5% methanol. When CH_3_OH was injected at 600 s, there was a sharp drop immediately in the curve of the 20 wt% Pt/C, and its relative current density decreased to 0.406 as shown in Figure 6b. However, the Ni/rGO-450 curve remained unchanged, and the relative current density was 0.893, indicating that it had excellent methanol resistance.

**Figure 7:**
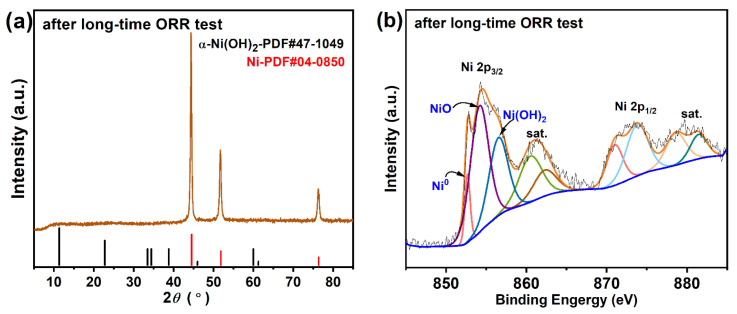
(**a**) XRD pattern and (**b**) Ni 2p spectra of Ni/rGO-450 catalyst after long-time ORR test.

The durability of the Ni/rGO-450 and 20 wt% Pt/C catalysts was evaluated by accelerated stress testing (AST) (Figure 6c,d). After 5000 cycles of CV, the E_1/2_ of Ni/rGO-450 was negatively shifted by only 47 mV (Figure 6d) which is smaller than that of Pt/C (74 mV, Figure 6c), indicating excellent electrochemical durability. Furthermore, the reason for the high ORR stability and durability of Ni/rGO-450 was explored by analyzing the phase and elemental valence state of Ni/rGO-450 before and after the ORR stability test. As illustrated by Figure 7a, the metallic Ni phase retained its characteristic diffraction peak in Ni/rGO following the long-term ORR test, and no NiO diffraction peak was detected. In Figure 7b, Ni still exhibited metal nickel and Ni^2+^ valence states. All these findings demonstrate that Ni/rGO-450 retains a stable structure after a prolonged ORR test. The outstanding long-term stability and durability of Ni/rGO-450 as an electrocatalyst for ORR resulted from integrating Ni atoms onto the rGO matrix (Ni/rGO), alleviating the loss of metallic species during the ORR [16]. As shown in Table S1, the ORR efficacy of the Ni/rGO electrocatalyst synthesized in this study surpasses that of other non-noble metal catalysts.

### 3.3. Mechanism towards Oxygen Reduction Reaction for Ni/rGO

**Figure 8:**
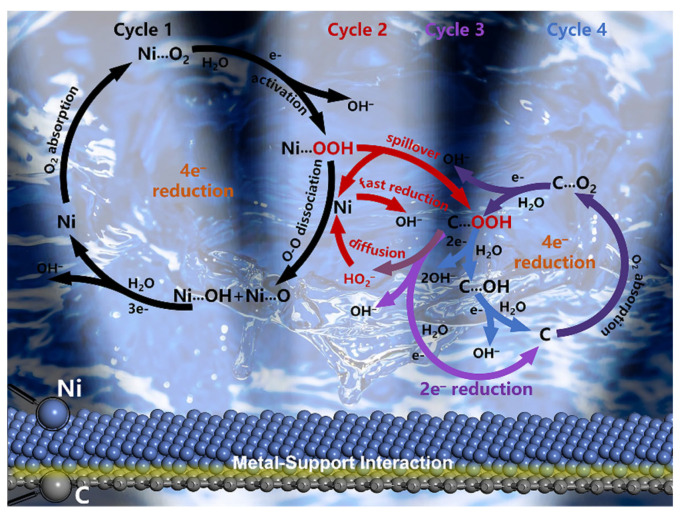
Schematic illustrating the proposed mechanism towards oxygen reduction reaction for nickel/reduced graphene oxide composite.

The possible ORR catalytic mechanism and pathway of the Ni/rGO electrocatalyst were proposed, as displayed in Figure 8. Here, in an alkaline environment, oxygen undergoes direct reduction to OH- (cycle 1) through a four-electron process that prevails on the nickel surface. The primary reaction equations are presented in Equations (S5)–(S8) in the Supplementary File. The ORR on graphene can be performed by 2e^−^ (Cycle 3) or 4e^−^ (Cycle 4) reduction, depending on the relative stability of the OOH* intermediate produced after O2 adsorption on the Ni/rGO catalyst. The loaded Ni NPs could activate its adjacent C sites [18]. Consequently, graphene is capable of reducing the chemical adsorption (Cycle 4 and Cycle 2) of ORR intermediates onto the nickel surface through the solid-phase spillover effect, allowing the continual regeneration of active sites on the Ni surface for adsorption and activation of other oxygen-containing species or O_2_ molecules from the KOH solution [54]. The HO_2_^−^ intermediates generated on the surface of graphene by 2e^−^ reduction can also efficiently diffuse towards the nearby Ni surface or the interface of Ni/rGO, where they can be further rapidly reduced to OH^−^ (Cycle 2 and Cycle 3). This mechanism acts as the pseudo-four-electron path on the RRDE, as the peroxide is rapidly reduced before detection by the ring electrode [55]. This method is extensively utilized in carbon-based electrocatalytic material systems to enhance four-electron pathway reactions on the surface of non-noble transition metals [56].

Here, metal–support interactions (MSI) played a role in altering catalytic activity. This resulted in a better mass transfer rate on the surface of the Ni NPs and increased utilization of the catalyst center due to higher migration and surface diffusion of active materials towards the Ni catalyst center [57]. The metallic characteristics of Ni NPs cause the electrons to have a mobility related to the nano-system, because the smaller the Ni NPs, the stronger the localization of their electronic states. MSI usually modulates the electronic characteristics of the supported metals by adjusting the occupation state of the metal d orbitals [7]. For an active metal surface, altering the d orbital will change its binding energy with the reaction intermediate. Consequently, this will influence the adsorption, activation, and desorption behavior of the reaction intermediate [10]. Although the intensity of EMSI in metals supported by carbon material is lower than that of metals supported by metal oxide, the interaction with the support must not be too strong, otherwise it will hinder the fine-tuning of the properties of the metal phase and may even affect the ORR activity [18].

**Figure 9:**
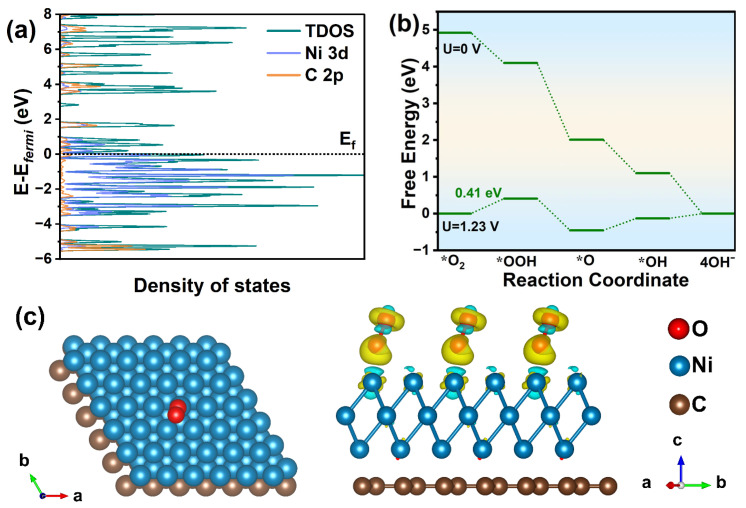
(**a**) Calculated DOS and PDOS results of Ni/rGO composite and (**b**) the calculated free energy diagrams of ORR process for Ni/rGO; (**c**) charge density difference of *O_2_ intermediates (RDS) on the surface of Ni/rGO. The yellow and cyan contours represent charge accumulation and depletion, respectively, in the real space with an isosurface level of ±0.002 |e| Bohr3.

Density functional theory (DFT) calculations were carried out to explain the catalytic properties and to explore the potential reaction mechanism that greatly improves the intrinsic activity after modifying Ni nanoparticles on the rGO support. Figure S7c shows that the Fermi level is still at the intersection between the π band and the π∗ band, compared to the band structure of graphene (Figure S7b). The conduction band passed through the Fermi level, which was due to the recombination of nickel atoms. However, the bandgap was still zero, indicating that the construction between Ni and graphene did not change the zero-bandgap property of graphene. Figure 9a shows that the Ni(111)/graphene interface is no longer mainly contributed by C atoms of graphene but is instead mainly contributed by Ni atoms. In addition, the PDOS of Ni 3d exhibited strength near the Fermi level (Ef), thereby improving the conductivity and electron transfer promotion of the composite [18]. Another parameter, the work function (Wf), denotes the minimum energy required for electrons to escape from the interior of a solid to the vacuum outside its surface. This can be utilized to assess the charge transfer between a metal and a support, the direction and extent of which are determined by the difference in Fermi levels of the metal NPs and the support, eventually reaching the equilibrium of the electron chemical potential. When the Wf of the metal exceeds that of the support, electrons transfer from the support to the metal and vice versa [10]. Figure S7d–f shows that the calculated Wf of monolayer rGO was 4.68 eV, and the Wf of Ni(111) was 5.25 eV. The calculated Wf of Ni/rGO (4.75 eV) was intermediate. The Ni has a larger work function than the rGO support, resulting in electron flow from the graphene to the Ni NPs. This electron transfer at the interface could potentially contribute to the significantly increased electrocatalytic efficiency of ORR.

Furthermore, to ascertain the rate-determining step (RDS) and their limiting reaction barrier during the ORR process, the free energies of each step in Ni/rGO models are calculated at two states, namely zero electrode potential (U = 0 V) and equilibrium potential (U = 1.23 V), as illustrated in Figure 9b. For analysis, oxygenated intermediates including *OOH, *O, and *OH species are considered, where * denotes the adsorption site. For the Ni/rGO, the first ORR electron transfer step (from O2 to *OOH) is regarded as the RDS, with an RDS energy barrier of 0.41 eV, which is much lower than that for Pt(111) whose overpotential is about 0.45 V (theoretical value) [58] or 0.44 V (experimental value) [59,60], indicating faster kinetics. Moreover, charge density difference analysis is conducted to investigate the electron transfer and structural interactions. As shown in Figure S7a, graphene with a π-electron system formed an interface with metallic nickel, where the difference in charge density illustrated electron transfer from graphene to Ni and subsequent accumulation at the interface. Concurrently, the structural model with adsorbed *O_2_ intermediate displays electron transfer from the Ni atoms to the adsorbed O_2_ molecules (Figure 9c), which contributes to reducing the RDS energy barrier.

In addition, the interface is in direct contact with Ni NPs, rGO, and reactants, which simultaneously promotes the catalytic reaction. DFT calculations have proved that atoms at the interfacial sites are conducive to the accumulation of excess charges. All this can significantly enhance the adsorption and reaction of molecules at the interfacial sites. Meanwhile, rGO is chemically inert to some extent. It can prevent the oxidation of transition metal nickel even when the loading is low, whereas this is an inevitable outcome when the nickel is supported on chemically active surfaces like oxides [61]. In conclusion, the presented Ni/rGO composite, prepared by a microwave-assisted hydrothermal method with calcination process control, demonstrates excellent ORR performance due to the synergistic effect, interfacial effect, and MSI of Ni and rGO. As a non-noble metal catalyst, it exhibits great potential as a cathode catalyst for fuel cells or metal–air batteries.

## 4. Conclusions

To sum up, graphene-supported nickel composites (Ni/rGO) were obtained by adjusting the pyrolysis temperature of the precursor and used as highly active ORR electrocatalysts in alkaline solutions. Experimental and theoretical results indicated that there was a metal–support interaction in the Ni/rGO composites. Electrons were redistributed on the nickel by interfacial charge transfer, and the electronic properties of the loaded nickel were modulated, thus affecting the catalytic activity. The limit diffusion current density of the optimum as-prepared Ni/rGO catalyst was 4.04 mA·cm^−2^ and its half-wave potential was 0.800 V. In addition, the four-electron reaction pathway of Ni/rGO was enhanced by the solid-phase spillover effect and the pseudo-four-electron mechanism. Meanwhile, rGO as the support can avoid the oxidation of transition metal Ni and improve the stability and anti-poisoning of the composite catalyst. The relative current density of the Ni/rGO-450 catalyst was 0.835 after testing for 20,000 s, and the relative current density was still 0.893 within 1000 s after methanol poisoning, both of which were better than the corresponding test parameters of 20 wt% Pt/C. After accelerated stress testing (AST), the E_1/2_ of Ni/rGO-450 was negatively shifted by only 47 mV, which is smaller than that of Pt/C (74 mV), indicating its excellent electrochemical durability. This work provides design strategies for the synthesis of highly active non-noble metal catalysts for energy conversion and storage, supporting their widespread use in industrial applications.

## Figures and Tables

**Table 1:** Particle size of Ni nanoparticles of different samples.

Samples	Specific Surface Area (m^2^ g^−1^)	Ni NPs Size (nm)	ln*D*	1/T (K^−1^)
Precursor	97.182	─	─	─
Ni/rGO-350	63.913	16.90318	2.8275	0.0016
Ni/rGO-450	74.545	21.60161	3.07277	0.00138
Ni/rGO-550	34.024	26.30211	3.26965	0.00121
Ni/rGO-650	26.307	29.52183	3.38513	0.00108
Ni/rGO-800	11.500	32.90283	3.49356	0.00093

## Data Availability

Data are contained within the article and Supplementary Materials.

## Supplementary Materials

The following supporting information can be downloaded at: https://www.mdpi.com/article/10.3390/nano13243087/s1, Figure S1: SEM images of rGO and precursor; Figure S2: N_2_ adsorption–desorption isotherm of (a) precursor, (b) Ni/rGO-350, (c) Ni/rGO-450, (d) Ni/rGO-550, (e) Ni/rGO-650, and (f) Ni/rGO-800 catalysts. The inset is the corresponding BJH pore size distribution curve; Figure S3: Relationship between lnD–1/T calcinated at different temperatures; Figure S4: (a) XPS survey and (b) O 1s of precursor and Ni/rGO composite at different calcination temperatures; Figure S5: (a) CV curves of the Ni/rGO catalysts with different calcination temperatures in O_2_- (solid line) and N_2_-saturated (dot line) 0.1 mol L^−1^ KOH at a scan rate of 50 mV s^−1^ and (b) LSV curves of Ni/rGO with different calcination temperatures in O_2_-saturated 0.1 mol L^−1^ KOH solution at a scan rate of 10 mV s^−1^ with different rotating speeds; Figure S6: (a) CV curves of the 20 wt% Pt/C catalysts in O_2_- (solid line) and N_2_-saturated (dot line) 0.1 mol L^−1^ KOH at a scan rate of 50 mV s^−1^ and (b) LSV curves of 20 wt% Pt/C catalysts in O_2_-saturated 0.1 mol L^−1^ KOH solution at a scan rate of 10 mV s^−1^ with different rotating speeds; Figure S7: (a) Structure of the interface formed between Ni(111) and rGO and the corresponding calculated charge density difference, where yellow and blue represent electron accumulation. The blue and brown spheres represent Ni and C atoms, respectively; Band structure of (b) rGO and (c) Ni/rGO; Work function of (d) rGO, (e) Ni(111), and (f) Ni(111)/rGO; Table S1: Comparison of various non-noble metal composites as ORR catalysts (References [62,63,64,65,66,67,68] are cited in the Supplementary Materials).
