# Supplementary material for: Facile Fabrication of Nickel Supported on Reduced Graphene Oxide Composite for Oxygen Reduction Reaction
Source: Nanomaterials (Basel). 2023 Dec 5;13(24):3087. doi: 10.3390/nano13243087 (PMC10745967; doi:10.3390/nano13243087)
Supplement: Supplementary file 1 [file nanomaterials-13-03087-s001.zip › nanomaterials-2729007-supplementary.pdf]

Supplementary File

Materials and Preparation process of Ni/rGO

All chemicals nickel nitrate ($Ni(NO_3)_2{\cdot}5H_2O$, 98.0%), tartaric acid (TA, $C_4H_6O_6$, 99.5%), urea ($(NH_2)_2CO$, 99.0%), ethylene glycol ($HOCH_2CH_2OH$, 99.0%), methanol(CH_3OH, 99.0%) and potassium hydroxide (KOH, 85.0%) were purchased from Tianjin DAMAO Chemical Reagent Co., Ltd (Tianjin, China) and used as received, except for graphene oxide (GO) (provided by Nanjing XFNANO Materials Tech Co., Ltd. (Nanjing, China)) and 5% Nafion solution (obtained from Shanghai HESEN Tech Co., Ltd. (Shanghai, China)). All the used water was RO water from UPK-I-10T Ulupure Ultra-pure water machine.

In a typical synthesis process as shown in Figure 1, 10 mmol of $Ni(NO_3)_2{\cdot}5H_2O$ and 5 mmol of tartaric acid were dissolved in 20 mL glycol, denoted as Solution A, with dissolving 50 mmol urea and 50 mg GO in 30 mL distilled water. Then, after ultrasonication for 30~60 min, the GO was fully dispersed in an aqueous solution, denoted as solution B. Solution A was then added slowly and uniformly to Solution B to obtain 50 mL of the glycol-water mixture (V_{glycol}/ V_{H_2O} =2:3). The as-obtained mixture was transferred to a 100 mL Teflon autoclave that connected with the microwave hydrothermal synthesizer (XH-300PE, XiangHu Technology) equipped with a temperature detector and pressure sensor. The mixtures were irradiated by microwave from room temperature to 180 °C in a gradient style, then heated for 60 min at 180 °C and finally cooled to 25 °C. Then the slurry in the reaction tank was centrifuged and stirred, and then was washed with distilled water and anhydrous ethanol three times, respectively, and was dried in the vacuum drying oven at 85 °C for 12 h.

The as-dried powder is the precursor. The precursor was pyrolyzed in the N_2 atmosphere after vacuuming in the tubular furnace. The temperature was increased from 50 °C to 450 °C at a rate of 5 °C min^{-1} and the calcination temperature was kept at 450 °C for 2 h. And then the calcined product was obtained after natural cooling.

Characterization methods

The microstructure and distribution of elements of the catalysts were investigated by Scanning electron microscopy (SEM, Hitachi SU8010) outfitted with an energy dispersive spectrometer (EDS). A JEOL JEM-2100F Transmission electron microscopy (TEM) was used to obtain high-resolution TEM images. Multi-point Brunauer-Emmett-Teller (BET) analysis was recorded on a Quantachrome Autosorb-IQ2-MP analyzer using N_2 adsorption at 77 K. Fourier transform infrared spectroscopy (FTIR) of the samples was obtained from Agilent Cary 660 FTIR. Thermogravimetry analysis (TG) was conducted on the TA Q600 Instruments from 25 ºC to 800 ºC under air at a heating rate of 10 $^{o}C \cdot min^{-1}$. X-ray diffraction (XRD) patterns were recorded using a Bruker D8 Advance diffractometer with Cu Kα radiation. Raman spectra of different catalysts were conducted on the Thermo Fisher DXR Raman Spectrometer equipped with an excitation source of 532 nm. A valence state analysis of elements was detected by Thermo Fisher Scientific ESCA-LAB Xi+ X-ray photoelectron spectroscopy (XPS).

Electrochemical characterization details

The electrochemical performance test of the obtained catalyst was carried out on a CHI760E electrochemical workstation (CH Instruments, China) using a standard three-electrode system with a rotating disk electrode (RDE) rotator instrument (RRDE-3A, ALS

Instrument Co.). A Hg/HgO electrode was used as the reference electrode (RE), a graphite rod as the counter electrode (CE) as the counter electrode, and a sample-modified rotating disk electrode (RDE) and a rotating ring disk electrode (RRDE) were the working electrodes(WE). The catalyst loading was 0.255 mg cm^{-2}.

Cyclic voltammetry (CV) was measured in the N_2- or O_2-saturated 0.1 mol L^{-1} KOH, with a scan rate of 50 mV s^{-1} at -0.8~0.2 V (vs. Hg/HgO). Linear sweep voltammetry (LSV) was carried out with rotating rates of 400~2500 rpm in the O_2-saturated 0.1 mol L^{-1} KOH, at a scan rate of 10 mV s^{-1}. The stability test was conducted using the chronoamperometric (I-t) test at -0.45 V (vs. Hg/HgO) in O_2-saturated 0.1 mol L^{-1} KOH. The electrochemical impedance spectroscopy (EIS) was recorded from 10^{-2} Hz to 10^{6} Hz at an open-circuit potential. All the resulting potentials were converted to the reversible hydrogen electrode (RHE) by the following Equation (S1):

$$E_{RHE} = E_{Hg/HgO} + E^{0}_{Hg/HgO} + 0.0591\times pH \quad (S1)$$

In this work, the RRDE measurements were carried out in a 0.1 M KOH solution under saturated oxygen conditions with a constant potential of 0.5 V (*vs.* Hg/HgO) on the ring electrode. The peroxide (HO_2^- in alkaline media) yield and the number of electrons transferred during the ORR reaction were calculated using the following Equations (S2):

$$\%HO_2^- = 200\times \frac{I_R/N}{I_D+(I_R/N)} \quad (S2)$$

$$n = \frac{4I_D}{I_D+(I_R/N)} \quad (S3)$$

Here, I_D is the disk current, I_R is the ring electrode current, N is the collection efficiency 0.424, HO_2^- % is the peroxide yield during the oxygen reduction reaction, and n is the number of electrons transferred per oxygen molecule.

Calculation details

Density functional theory (DFT) based first-principles calculations are performed using

the projected augmented wave (PAW) method implemented in the Vienna ab initio simulation package (VASP) [1]. The generalized gradient approximation (GGA) with Perdew Burke-Ernzerhof (PBE) functional and the projector augmented wave (PAW) potential were employed [2,3]. The kinetic energy cut-off was set to 450 eV. The Brillouin-zone (BZ) integration is carried out using the Monkhorst-Pack sampling method with a density of 3×3×1 for simulations. A sufficiently large vacuum region of 15 Å was used for all the systems to ensure the periodic images to be well separated. The convergence criterion of force and energy were set to 0.02 eV $Å^{-1}$ and 10^{-4} eV, respectively. The ORR pathways on the considered models were calculated in details according to electrochemical framework developed by Norskov. The primary reaction equations are presented in Equation (2)~(5).

The catalytic activity of these catalysts was evaluated by calculating the change of Gibbs free energy (ΔG) of each elementary step in ORR reaction (presented in Equation (S5)~(S8)), based on the computational hydrogen electrode model proposed by Nørskov, which is expressed by the following equation:

$$\Delta G=\Delta E_{ads}+\Delta ZPE-T\Delta S-eU-kBTln(10)\times pH \quad (S4)$$

The primary reaction equations are presented in Equation (2)~(5):

$$* + O_2 + H_2O + e^- \rightarrow HOO^* + OH^- \quad (S5)$$

$$HOO^* + e^- \rightarrow O^* + OH^- \quad (S6)$$

$$O^* + H_2O + e^- \rightarrow HO^* + OH^- \quad (S7)$$

$$HO^* + e^- \rightarrow * + OH^- \quad (S8)$$

where ΔE_{ads}, ΔZPE, T, ΔS, eU, kBTln(10)×pH are adsorption energy, zero-point energy, temperature, entropy change, change of free energy due to applied potential on electrode, the correction of the H^+ free energy resulted from the acidity and alkalinity of solution.

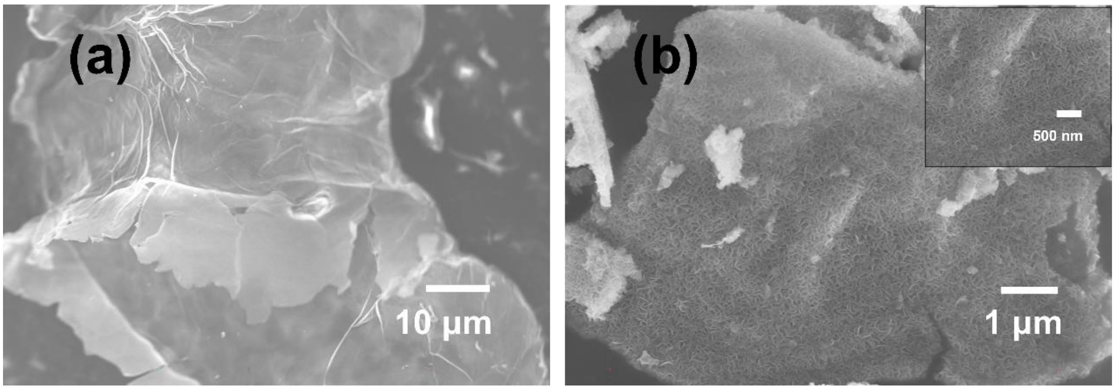

Figure S1. SEM images of rGO and precursor

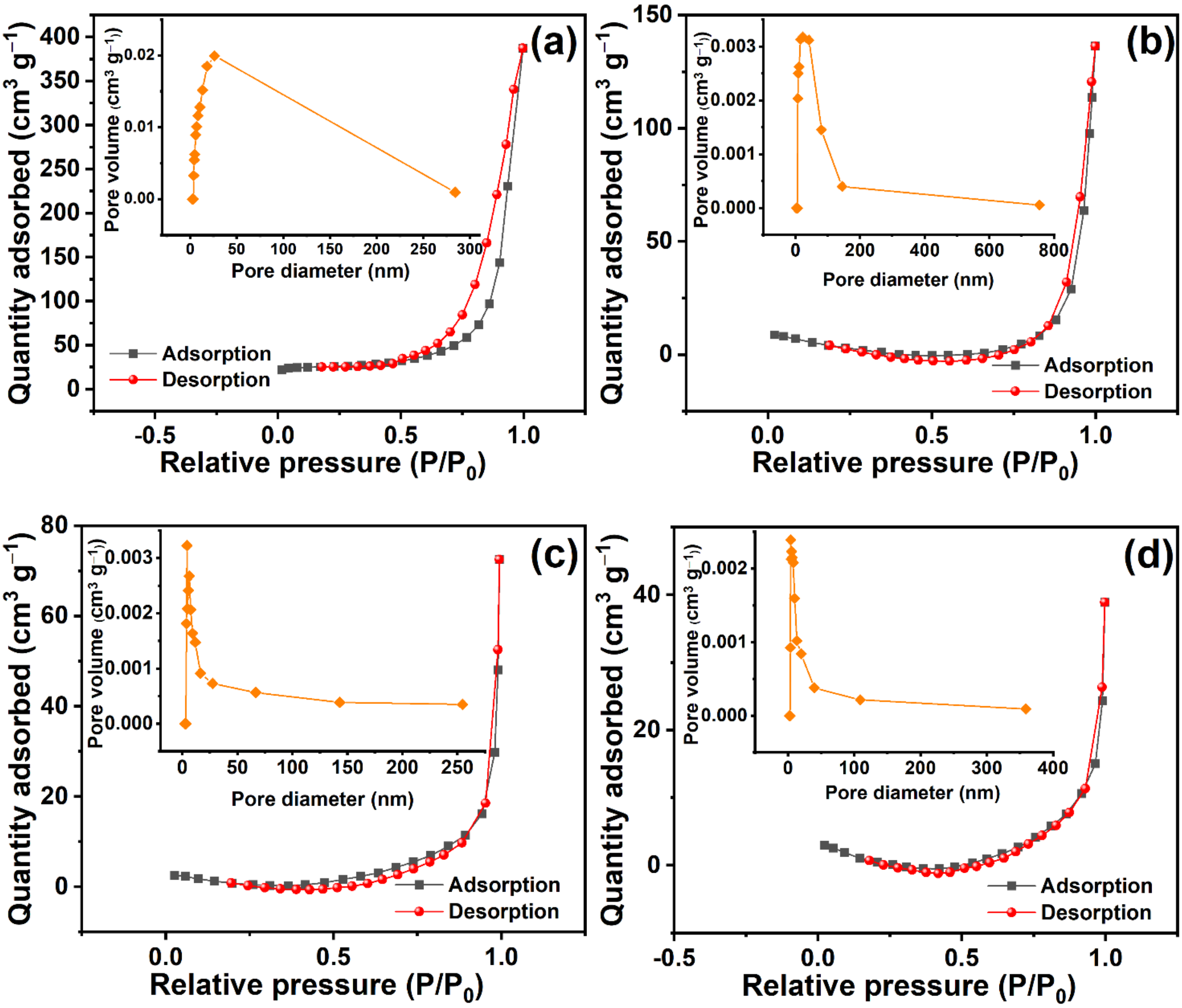

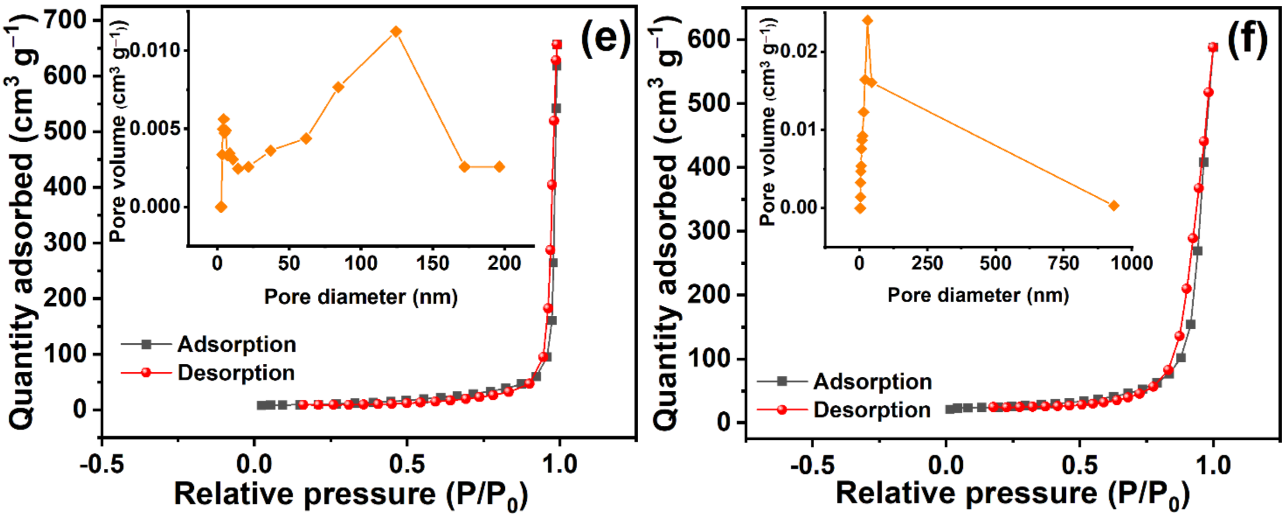

Figure S2. N_2 adsorption-desorption isotherm of (**a**) precursor, (**b**) Ni/rGO-350, (**c**) Ni/rGO-450, (**d**) Ni/rGO-550, (**e**) Ni/rGO-650 and (**f**) Ni/rGO-800 catalysts. The inset is the corresponding BJH pore size distribution curve.

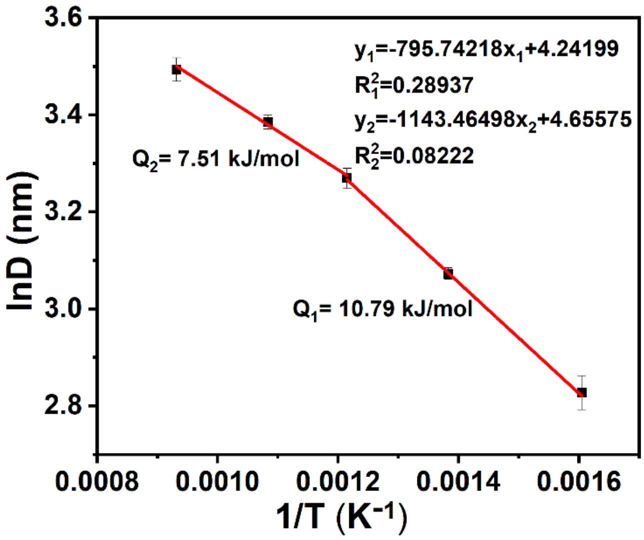

Figure S3. Relationship between lnD-1/T calcinated at different temperatures.

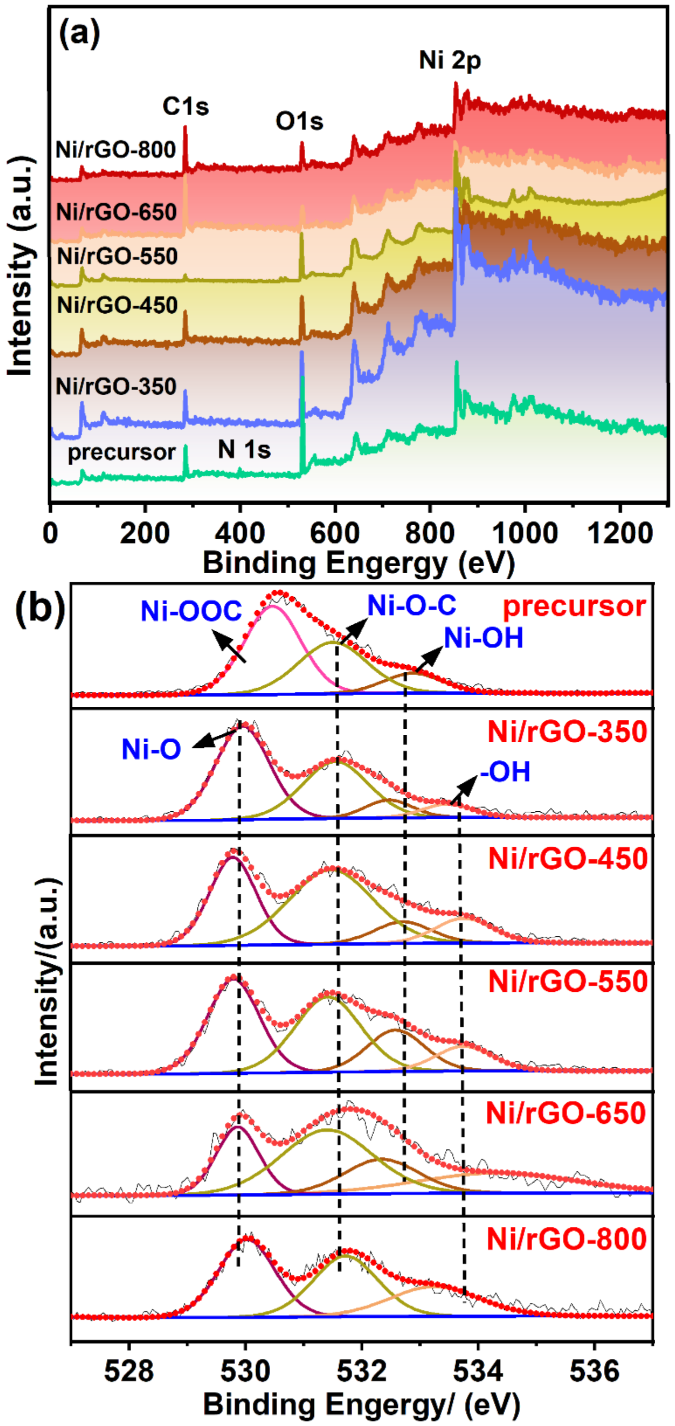

Figure S4. (**a**) XPS survey and (**b**) O 1s of of precursor and Ni/rGO composite under different calcination temperature.

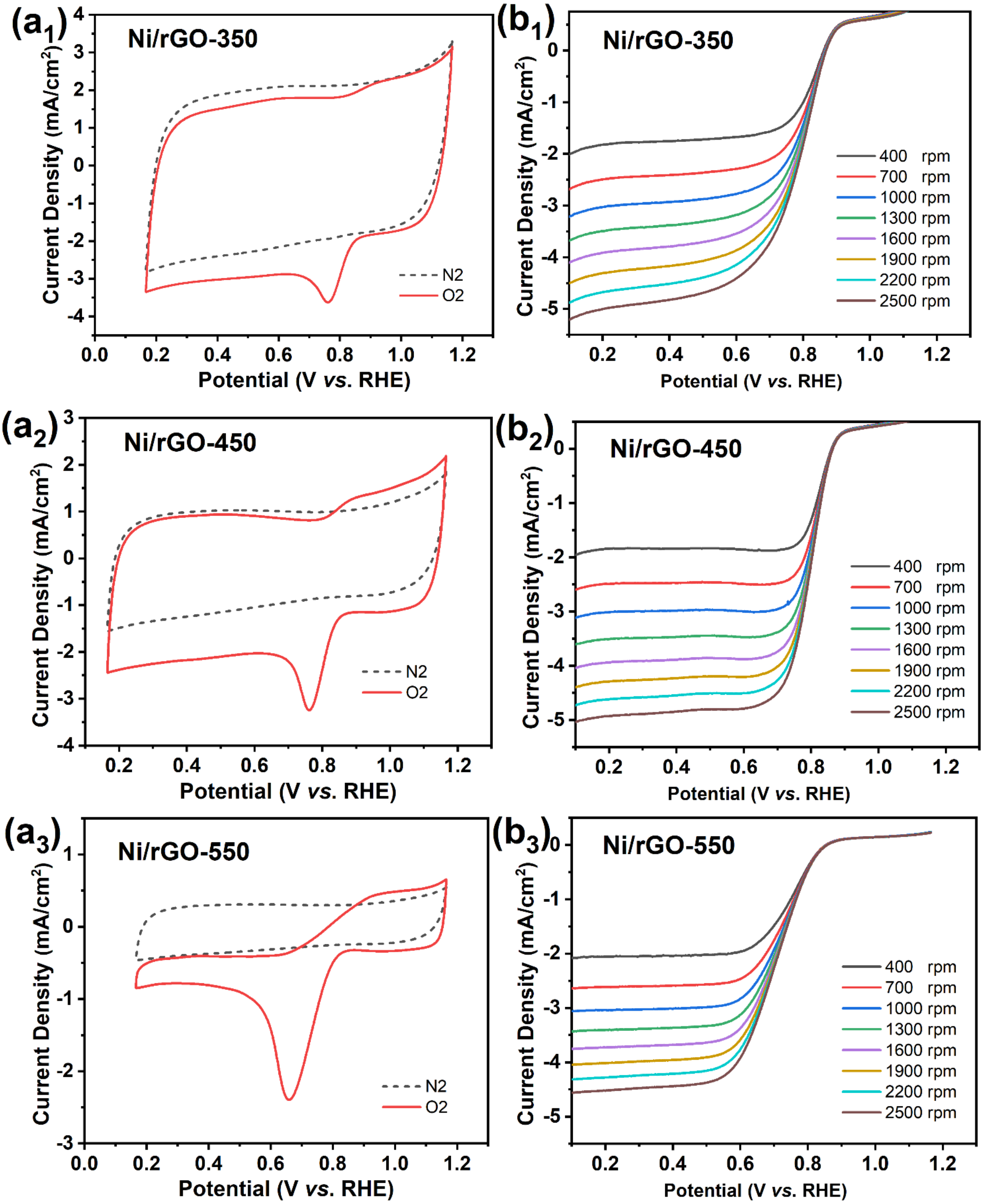
(a1)
Ni/rGO-350
Current Density (mA/cm2)
Potential (V vs. RHE)
N2
O2
(b1)
Ni/rGO-350
Current Density (mA/cm2)
Potential (V vs. RHE)
400 rpm
700 rpm
1000 rpm
1300 rpm
1600 rpm
1900 rpm
2200 rpm
2500 rpm
(a2)
Ni/rGO-450
Current Density (mA/cm2)
Potential (V vs. RHE)
N2
O2
(b2)
Ni/rGO-450
Current Density (mA/cm2)
Potential (V vs. RHE)
400 rpm
700 rpm
1000 rpm
1300 rpm
1600 rpm
1900 rpm
2200 rpm
2500 rpm
(a3)
Ni/rGO-550
Current Density (mA/cm2)
Potential (V vs. RHE)
N2
O2
(b3)
Ni/rGO-550
Current Density (mA/cm2)
Potential (V vs. RHE)
400 rpm
700 rpm
1000 rpm
1300 rpm
1600 rpm
1900 rpm
2200 rpm
2500 rpm

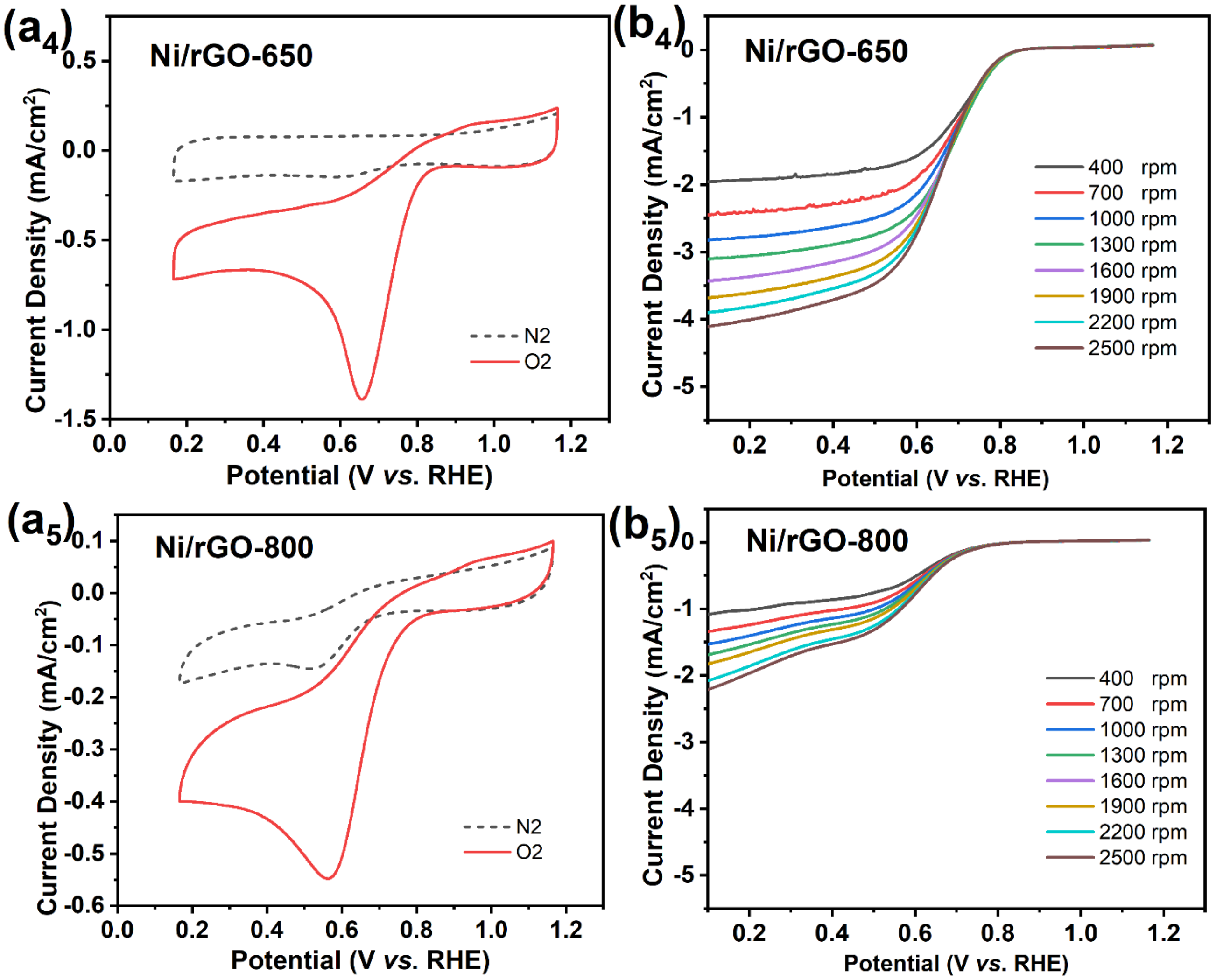

Figure S5. (**a**) CV curves of the Ni/rGO with different calcination temperature catalysts in O_2-(solid line) and N_2-saturated (dot line) 0.1 mol L^{-1} KOH at a scan rate of 50 mV s^{-1} and (**b**) LSV curves of Ni/rGO with different calcination temperature in O_2-saturated 0.1 mol L^{-1} KOH solution at a scan rate of 10 mV $s^{-1}$with different rotating speeds.

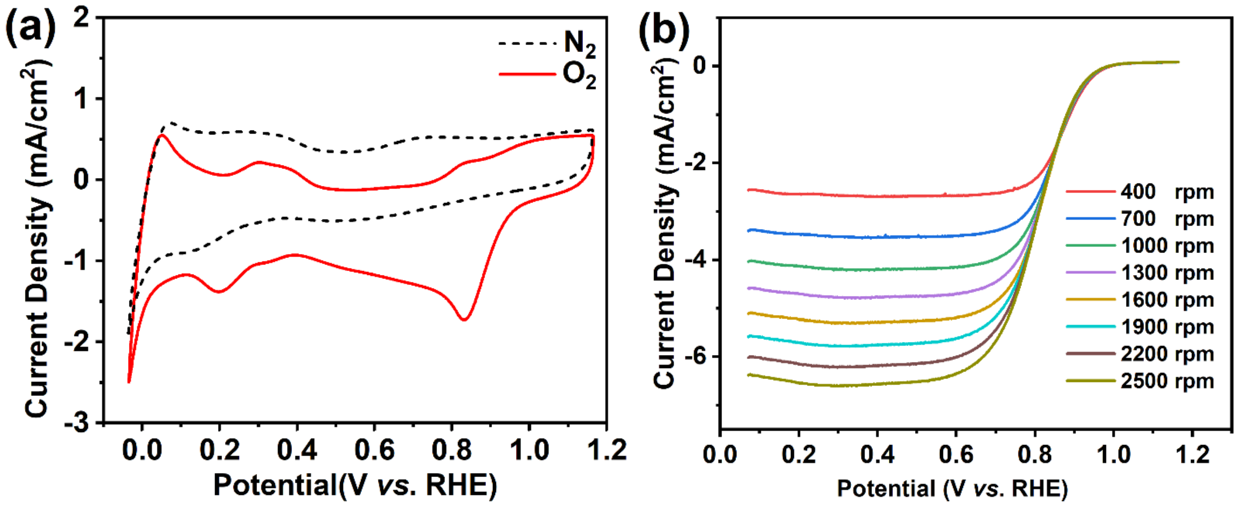

Figure S6. (**a**) CV curves of the 20wt% Pt/C catalysts in O_2-(solid line) and N_2-saturated (dot line) 0.1 mol L^{-1} KOH at a scan rate of 50 mV s^{-1} and (**b**) LSV curves of 20wt% Pt/C catalysts in O_2-saturated 0.1 mol L^{-1} KOH solution at a scan rate of 10 mV $s^{-1}$with different rotating speeds.

Table S1. Comparison of various non-noble metal composites as ORR catalysts.

Catalyst	E_{onset} *vs* RHE(V)	$E_{1/2}$ *vs* RHE(V)	j_L (mA cm^{-2})	Stability	Ref
$Cu_{0.31}Ni_{0.69}O$/rGO	0.652	0.472	1.20	After 16000 s J/J_0=0.920	[4]
CoOx/CNTs	0.657	0.577	1.60	After 2000 s, J/J_0=0.900	[5]
CuO/N-doped rGO	1.063	-	3.063	After 5000 s, J/J_0=0.925	[6]
$NiCo_2O_4$-rGO	0.830	-	3.87	After 5000 cycles, a negative shift of only 18 mV for $E_{1/2}$	[7]
**Ni/rGO**	**0.864**	**0.800**	**4.04**	**After 20000 s, J/J_0=0.835**	**This work**

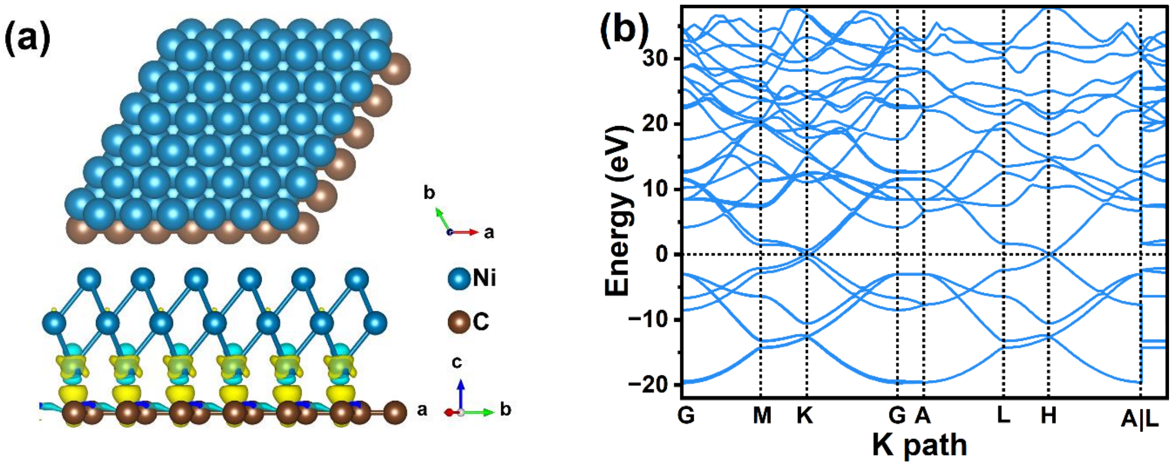

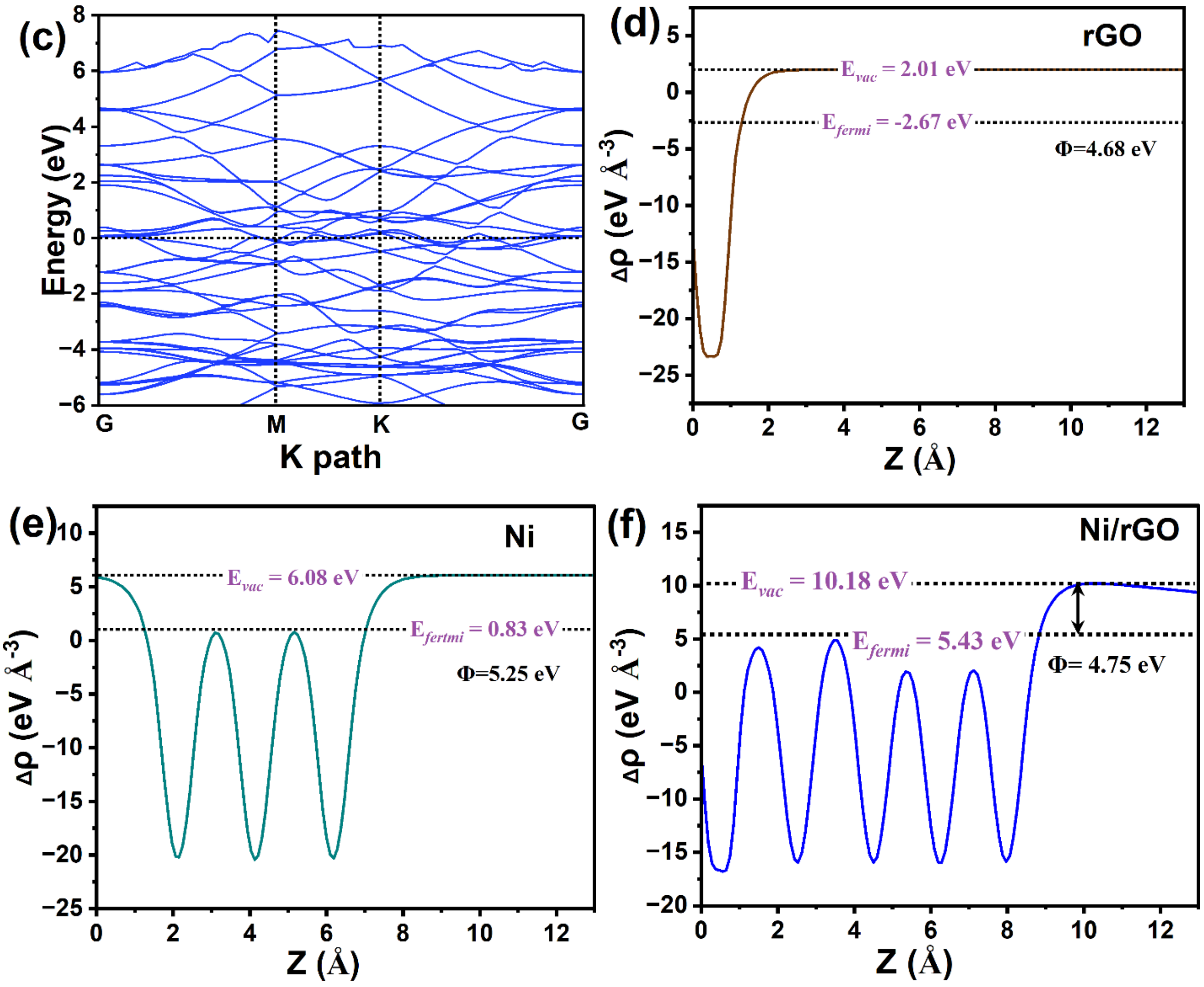

Figure S7. (**a**) Structure of the interface formed between Ni (111) and rGO, and the corresponding calculated charge density difference, where yellow and blue represent electron accumulation. The blue and brown spheres represent Ni and C atoms, respectively; Band structure of (**b**) rGO and (**c**) Ni/rGO; Work function of (**d**) rGO, (**e**) Ni(111) and (**f**) Ni(111)/rGO

Reference

[1] G. Kresse, J. Hafner, Ab initio molecular dynamics for liquid metals, Physical Review B 47 (1993) 558-561, https://doi.org/10.1103/PhysRevB.47.558.

[2] G. Kresse, D. Joubert, From ultrasoft pseudopotentials to the projector augmented-wave method, Physical Review B 59 (1999) 1758-1775, https://doi.org/10.1103/PhysRevB.59.1758.

[3] J.P. Perdew, K. Burke, M. Ernzerhof, Generalized Gradient Approximation Made Simple, Physical Review Letters 77 (1996) 3865-3868, https://doi.org/10.1103/PhysRevLett.77.3865.

[4] J. Yu, Z. Jiang, J. Wang, H. Fang, T. Huang, S. Sun, Synthesis of high performing $Cu_{0.31}Ni_{0.69}O$/rGO hybrid for oxygen reduction reaction in alkaline medium, International Journal of Hydrogen Energy 44 (2019) 13345-13353, https://doi.org/10.1016/j.ijhydene.2019.03.215.

[5] A.B.A.A. Nassr, T. Kottakkat, M. Bron, A simple microwave process for the preparation of cobalt oxide nanoparticles supported on carbon nanotubes for electrocatalytic applications, Journal of Solid State Electrochemistry 24 (2020) 131-136, https://doi.org/10.1007/s10008-019-04477-1.

[6] L. Zhang, J. Lu, J. Wei, Y. Wang, Novel flower-like CuO/N-rGO as enhanced electrocatalyst for oxygen reduction reaction, Nano 14 (2019) 1950132, https://doi.org/10.1142/S1793292019501327.
[7] Z.-Y. Wu, P. Chen, Q.-S. Wu, L.-F. Yang, Z. Pan, Q. Wang, Co/Co_3O_4/C–N, a novel nanostructure and excellent catalytic system for the oxygen reduction reaction, Nano Energy 8 (2014) 118-125, https://doi.org/10.1016/j.nanoen.2014.05.019.
